# Screw Impingement Causing Massive Swelling of the Sciatic Nerve: A Case Report

**DOI:** 10.7759/cureus.31059

**Published:** 2022-11-03

**Authors:** Abdelilah Rhoul, Mohammed Gartit, Mohamed Noumairi, Siham Elmir, Abdallah El-Sayed Allam, Adnane Lachkar, Najib Abdeljaouad, Hicham Yacoubi, Ahmed Amine EL Oumri

**Affiliations:** 1 Faculty of Medicine and Pharmacy, Mohammed First University, Oujda, MAR; 2 Department of Physical Medicine and Rehabilitation, Mohammed VI University Hospital, Oujda, MAR; 3 Morphological Madrid Research Center (MoMaRC), UltraDissection Spain EchoTraining School, Madrid, ESP; 4 Physical Medicine, Rheumatology, and Rehabilitation, Tanta University Hospitals, Faculty of Medicine, Tanta University, Tanta, EGY; 5 Department of Orthopedic Trauma, Mohammed VI University Hospital, Oujda, MAR

**Keywords:** sciatic nerve, nerve entrapment, peripheral nerve ultrasonography, hip dislocation, acetabulum fractures, sciatic nerve injury

## Abstract

Sciatic nerve (SN) injuries after hip fracture dislocation are described and are not uncommon. Several factors can lead to SN injury after hip surgery; among other factors, screw plates of synthesis materials can immigrate and lead to nerve impingement.

We report a case of a 22-year-old male with a history of posterior wall fracture and hip dislocation after a motorway accident. Ultrasonography showed massive swelling of the SN with a cross-sectional area measured at 1.50 cm^2^ upstream to screw impingement. The reoperation option was judged too risky by the orthopedic surgeons; currently, the patient is undergoing platelet-rich plasma (PRP) injections around the nerve swelling and to the lifter muscles of the foot.

## Introduction

A sciatic nerve (SN) injury after acetabular fractures or hip dislocation is not an unusual complication of these orthopedic emergencies [1]. Nerve injury can be a result of the initial trauma or an iatrogenic trauma during surgery and can also occur as a late complication of surgery [2]. Several studies showed that posterior wall fractures of the acetabulum were more associated with SN injury [3]. We report a new case of SN injury after fracture-dislocation of the hip. A screw impingement caused significant swelling of the SN.

## Case presentation

**Figure 1:**
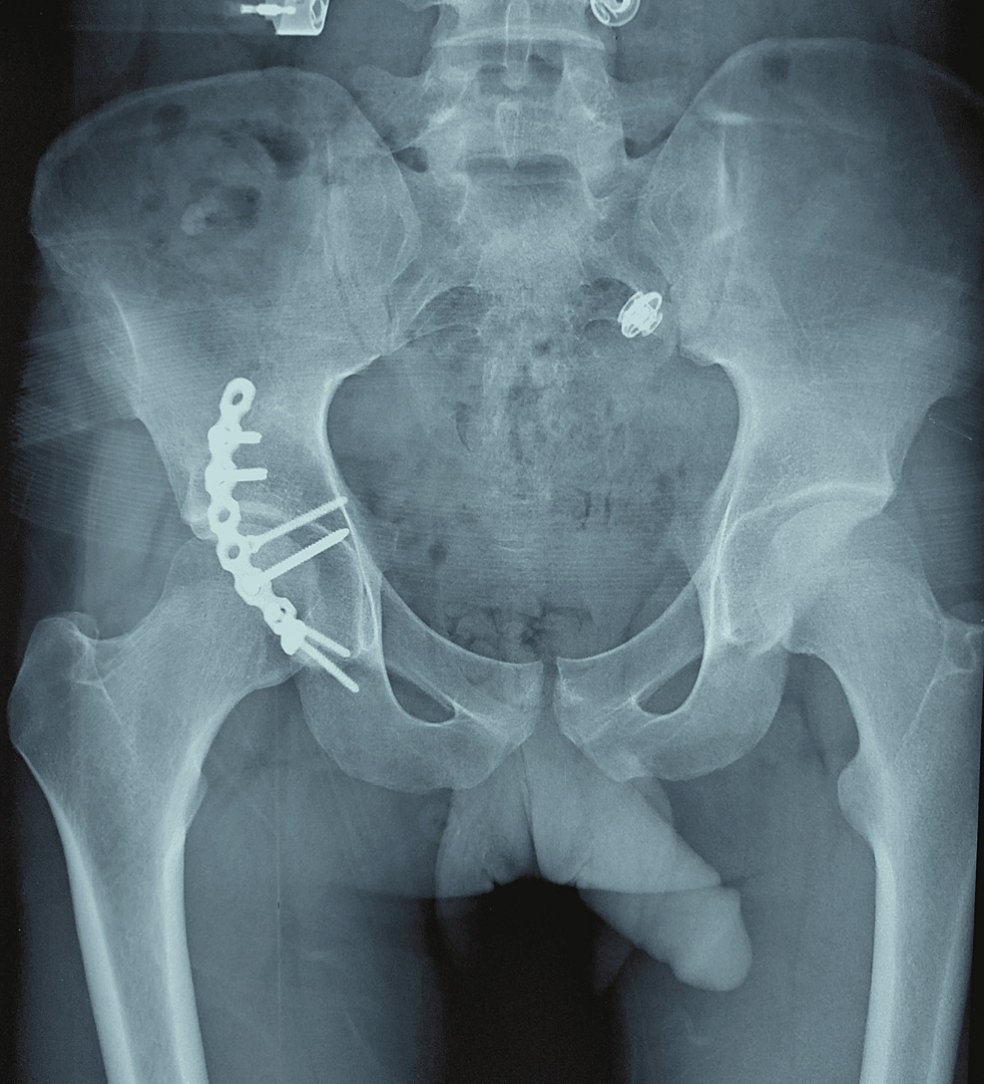
Postoperative pelvic X-ray showing the plate screw fixation to the posterior wall of the acetabulum.

A 22-year-old young man with no past medical history was hit by a car while driving his motorcycle two years ago. The physical examination at the emergency unit revealed a total function impotence of the right lower limb. Pelvic X-rays showed a right acetabulum fracture in addition to total hip dislocation. The patient was then transferred to the operating room and had a dislocation reduction with the insertion of two screws of 3.5 mm directly into the cortical bone and an installation of a curved plaque with two screws on both sides of the fractured fragment. Two pins were also temporarily fixed to the posterior wall of the acetabulum (Figure 1).

One year after his accident, the patient was addressed to our department of physical medicine and rehabilitation. The physical examination found a steppage gait of the right foot and the muscle strength was evaluated at 2/5 on dorsiflexion of the tight foot. The patient was unable to walk in his tales. In addition, the right knee-jerk reflex was absent, and the patient also presented hypoesthesia of the right leg.

**Figure 2:**
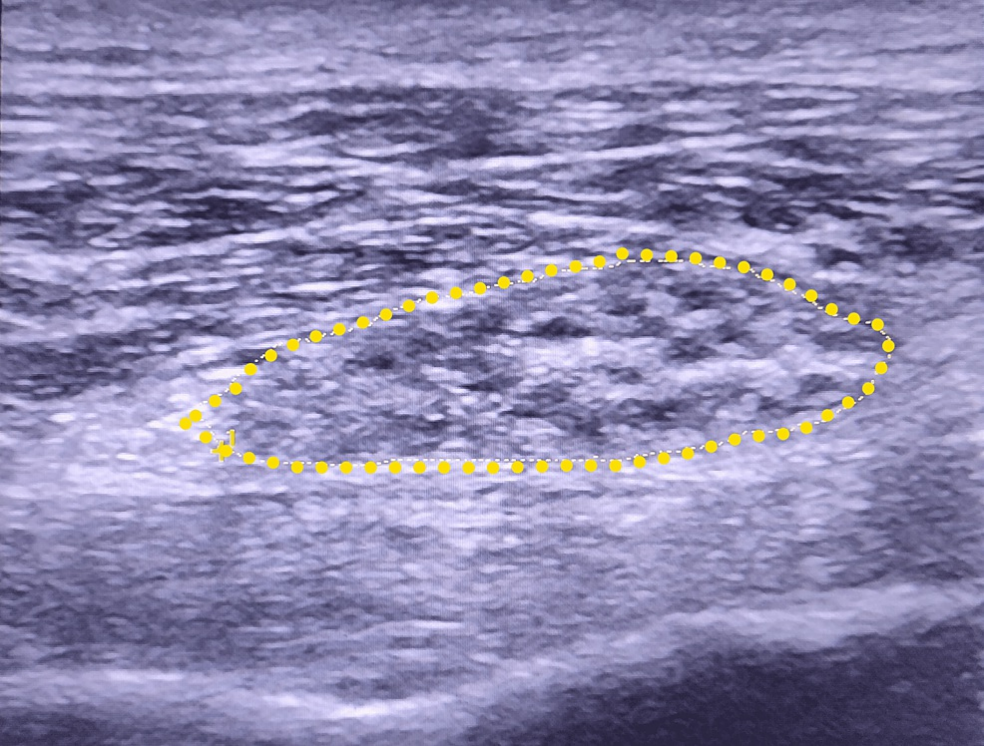
Short axis view of the injured sciatic nerve (yellow dotted line) with the cross-sectional area (CSA) measurement (1.50 cm2).

**Figure 3:**
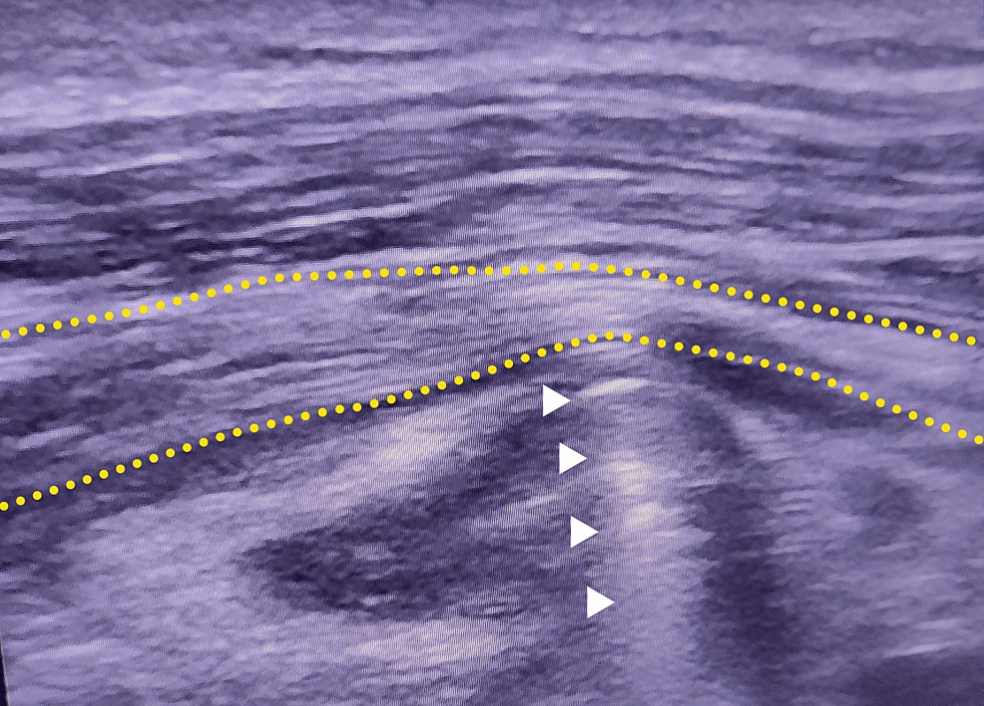
Long axis view of the injured sciatic nerve (yellow dotted lines) showing important swelling up down to screw entrapment (white arrowheads).

**Figure 4:**
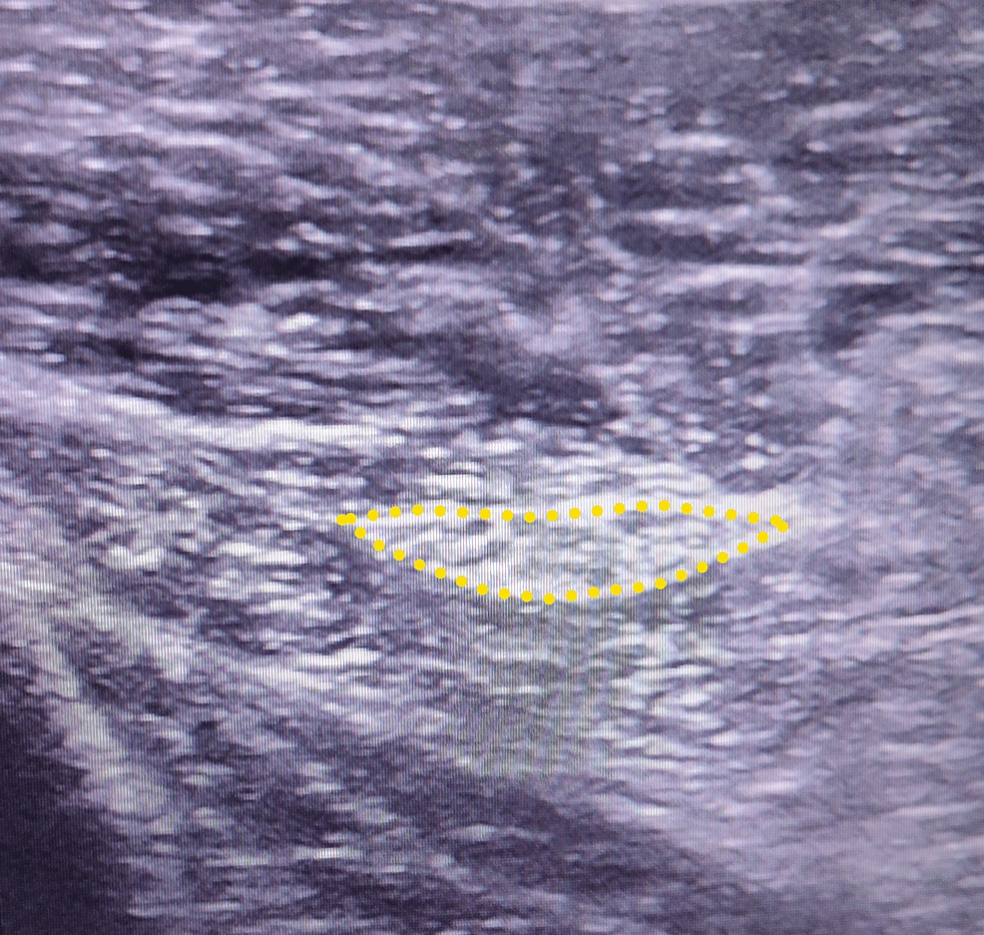
Normal sciatic nerve (yellow dotted line) of the contralateral limb with cross-sectional area (CSA) measurement (0.33 cm2).

Electromyography (EMG) of the lower limbs showed a severe truncal axonal injury of the common fibular nerve. Hip and SN ultrasonography showed an important swelling of the SN of the right limb. The cross-sectional area (CSA) of the injured nerve was very large at 1.50 cm^2 ^(Figure 2). The long axis view showed screw impingement on the SN, in addition to an important swelling of the SN upstream of the place in contact with the screw (Figure 3). The surface of the contralateral SN was also calculated, finding a measurement of 0.30 cm^2^ (Figure 4).

The decision of orthopedic surgeons was not favorable for reoperation and removing the screws, judging it as complicated and too risky for the patient. The patient is currently undergoing ultrasound-guided injections of platelet-rich plasma (PRP) around the nerve swelling and in the foot lifter muscles in addition to physical rehabilitation.

## Discussion

Several postoperative factors were described to be associated with SN injury [4]. A hematoma may produce compressive neuropathy in the first few days following surgery [5]. Later, causal events such as muscle or capsular scarring, the migration of osteosynthesis material, and heterotopic ossifications have been also identified [4,6,7]. From that, the anamnesis detailing the date of the onset of symptoms in relation to surgery and the physical examination data are both essential to find the right etiology of the SN swelling.

Ultrasonography provides good-quality morphological imaging of the SN with respect to its exact location, course, and extent [8,9]. The mean range of normal SN CSA was 0.47-0.59 cm^2^ at the level of the gluteal sulcus and 0.39-0.50 cm^2^ at the midpoint of the thigh in a normal population [10]. In our case, the CSA of the injured nerve was one if not the largest ever measured at 1.50 cm^2 ^(Figure 2).

Preventive measures

Preventive approaches during hip surgery have been recommended by numerous authors, especially with the posterior approach such as keeping the hip extended and the knee flexed to minimize traction on the SN [11]. Furthermore, the retractors should be carefully positioned so that extensive posterior retraction with the hip in flexion will be avoided [2]. Otherwise, intraoperative SN monitoring with somatosensory evoked potentials (SSEPs) and spontaneous EMG may be helpful to prevent nerve injury, particularly with high-risk acetabular fractures [12].

Murena et al. conducted a systematic review, evaluating the effectiveness of intraoperative nerve monitoring (IONM) in hip and pelvis orthopedic and trauma surgery. The results were insufficient to support the routine use of IONM. Nevertheless, IONM could be helpful in critical maneuvers, positions, or pathologies that could lead to SN intraoperative damage [13].

Sciatic nerve decompression

SN surgical decompression or reconstructive surgery after acetabulum fractures were reported. The results showed better results on sensory neuropathy than the motor one [14-16]. Recently, a retrospective study assessing the endoscopic SN decompression after a fracture or reconstructive surgery of the acetabulum in comparison with endoscopic treatments in idiopathic deep gluteal syndrome showed some degree of relief after the endoscopic SN release [11]. In addition, three patients with motor weakness and without foot drop showed complete motor improvement. However, complete motor improvement was not seen in patients with foot drop [11].

In our case, reoperation was considered too risky for the patient. Ultrasound-guided injections of PRP around the nerve and in the lifter muscles of the injured foot were proposed for the patient. Indeed PRP is believed to provide essential growth factors to promote repair in tissues with low healing potential [17]. Reports of PRP injections to treat peripheral nerve injury are mainly based on animal experiments and in vitro studies with promising results [18,19]. Clinical application reports, although rare, showed encouraging results for nerve regeneration treated with PRP [20,21].

Another possible therapy in this condition could be ozone therapy (OT). This medical therapy consists of delivering oxygen, with ozone comprising fewer than 5% at its highest concentration. This therapy was experimented on rats with SN injury [22]. Ozone was administered daily at a concentration of 35-40 ug/ml and volume of 5 ml by intraperitoneal method for two months. The results showed significant functional improvement after two and four weeks after OT [22]. Other experimental studies showed favorable results on nerve regeneration and reduction of myelin and axonal injuries after OT was used alone or in association with methylprednisolone [23,24]. Although these results are encouraging, further investigations and clinical trials are needed to assess the potential therapeutic role of OT.

## Conclusions

SN injury should be looked for after posterior wall fractures of the acetabulum and hip dislocation. The initial physical examination and clinical history are essential to determine the etiology of the nerve injury and preventable measures should be always taken to prevent it.
